# Commissural communication allows mouse intergeniculate leaflet and ventral lateral geniculate neurons to encode interocular differences in irradiance

**DOI:** 10.1113/JP276917

**Published:** 2018-10-23

**Authors:** A. Pienaar, L. Walmsley, E. Hayter, M. Howarth, T. M. Brown

**Affiliations:** ^1^ Faculty of Biology, Medicine and Health, School of Medicine University of Manchester Manchester UK

**Keywords:** vision, circadian, electrophysiology

## Abstract

**Key points:**

- Unlike other visual thalamic regions, the intergeniculate leaflet and ventral lateral geniculate nucleus (IGL/vLGN) possess extensive reciprocal commissural connections, the functions of which are unknown.
- Using electrophysiological approaches, it is shown that commissural projecting IGL/vLGN cells are primarily activated by light increments to the contralateral eye while cells receiving commissural input typically exhibit antagonistic binocular responses.
- Across antagonistic cells, the nature of the commissural input (excitatory or inhibitory) corresponds to the presence of ipsilateral ON or OFF visual responses and in both cases antagonistic responses disappear following inactivation of the contralateral thalamus.
- The steady state firing rates of antagonistic cells uniquely encode interocular differences in irradiance.
- There is a pivotal role for IGL/vLGN commissural signalling in generating new sensory properties that are potentially useful for the proposed contributions of these nuclei to visuomotor/vestibular and circadian control.

**Abstract:**

The intergeniculate leaflet and ventral lateral geniculate nucleus (IGL/vLGN) are portions of the visual thalamus implicated in circadian and visuomotor/vestibular control. A defining feature of IGL/vLGN organisation is the presence of extensive reciprocal commissural connections, the functions of which are at present unknown. Here we use a combination of multielectrode recording, electrical microstimulation, thalamic inactivation and a range of visual stimuli in mice to address this deficit. Our data indicate that, like most IGL/vLGN cells, those that project commissurally primarily convey contralateral ON visual signals while most IGL/vLGN neurons that receive this input exhibit antagonistic binocular responses (i.e. excitatory responses driven by one eye and inhibitory responses driven by the other), enabling them to encode interocular differences in irradiance. We also confirm that this property derives from commissural input since, following inactivation of the contralateral visual thalamus, these cells instead display monocular contralateral‐driven ON responses. Our data thereby reveal a fundamental role for commissural signalling in generating new visual response properties at the level of the visual thalamus.

## Key points

- Unlike other visual thalamic regions, the intergeniculate leaflet and ventral lateral geniculate nucleus (IGL/vLGN) possess extensive reciprocal commissural connections, the functions of which are unknown.
- Using electrophysiological approaches, it is shown that commissural projecting IGL/vLGN cells are primarily activated by light increments to the contralateral eye while cells receiving commissural input typically exhibit antagonistic binocular responses.
- Across antagonistic cells, the nature of the commissural input (excitatory or inhibitory) corresponds to the presence of ipsilateral ON or OFF visual responses and in both cases antagonistic responses disappear following inactivation of the contralateral thalamus.
- The steady state firing rates of antagonistic cells uniquely encode interocular differences in irradiance.
- There is a pivotal role for IGL/vLGN commissural signalling in generating new sensory properties that are potentially useful for the proposed contributions of these nuclei to visuomotor/vestibular and circadian control.

## Introduction

The intergeniculate leaflet and ventral lateral geniculate nucleus (IGL/vLGN) are related portions of the visual thalamus implicated in circadian and visuomotor/vestibular control (Harrington, 1997; Morin & Allen, 2006; Brown, 2016). At present, understanding of the roles and properties of IGL/vLGN neurons is relatively limited, by comparison to regions of the visual thalamus (in rodents the dorsal lateral geniculate; dLGN) subserving image‐forming visual perception (Usrey & Alitto, 2015).

Unlike dLGN neurons, most cells in the IGL/vLGN are GABAergic (Moore & Speh, 1993; Lein *et al*. 2007) and, in the case of the IGL, co‐express various neuropeptides (Moore & Card, 1994; Harrington, 1997; Morin & Blanchard, 2001) including neuropeptide Y (NPY) and met‐enkephalin (ENK). The IGL/vLGN also differs from dLGN in that they provide very extensive subcortical connections (Moore *et al*. 2000; Morin & Blanchard, 2005). Most notably, one characteristic feature is the presence of very robust commissural connections between the IGL/vLGN and their counterparts in the opposite hemisphere (Pickard, 1982; Cosenza & Moore, 1984; Nakamura & Kawamura, 1988; Mikkelsen, 1992; Park *et al*. 1993; Morin & Blanchard, 1995). By contrast, commissural connections between the dLGN are sparse or absent, suggesting commissural communication plays some specific role in IGL/vLGN function.

One hypothesis as to the function of commissural communication has been that it is involved in coordinating the slow rhythmic bursting often observed in visual nuclei in anaesthetised animals (Lewandowski & Błasiak, 2004; Szkudlarek *et al*. 2008; Cheong *et al*. 2011). However, a direct test of this hypothesis indicated that commissural input was not required for this oscillatory activity (Lewandowski *et al*. 2002), although it does appear to be a property exhibited by commissurally projecting IGL neurons in rats (Blasiak & Lewandowski, 2013).

An alternate hypothesis is that commissural signalling contributes to the sensory properties of IGL/vLGN neurons. Two studies using antidromic activation to identify commissurally projecting IGL neurons (Zhang & Rusak, 1989; Blasiak & Lewandowski, 2013) indicate that such cells generally show sustained light‐dependent increases in firing, in common with many other cells in the IGL/vLGN (Harrington & Rusak, 1989; Harrington, 1997; Thankachan & Rusak, 2005; Howarth *et al*. 2014). One of these earlier studies also provided evidence that intense stimulation of the IGL region generally suppressed activity in the contralateral IGL (Zhang & Rusak, 1989). As such, it has been suggested that commissural signalling may contribute to inhibitory binocular interactions in the IGL/vLGN. Indeed, we and others have previously found subpopulations of cells exhibiting antagonistic responses to stimulation of the ipsilateral *vs*. contralateral eye (Harrington & Rusak, 1989; Howarth *et al*. 2014) that are specifically enriched within these portions of the visual thalamus that receive extensive commissural input.

Here we set out to better define the functional significance of commissural communication in the IGL/vLGN and to test the hypothesis that this plays a role in generating antagonistic binocular interactions.

## Methods

### Ethical approval

All animal use was in accordance with the Animals (Scientific Procedures) Act 1986 (UK), received institutional ethics committee and UK Home Office approval, and conformed to the principles and standards set out by Grundy (2015). Wild‐type mice (C57Bl/6 background; bred in the Biological Services Facility, University of Manchester) were housed under a 12 h dark–light cycle environment at a temperature of 22°C with food and water *ad libitum*. Zeitgeber time (ZT) 0 was designated as the time of lights on. A total of 67 male mice (50–100 days old) were used for the experiments described below. Animals were removed from their home cage during the early portion of the light phase (ZT 1–3) and anaesthetised by i.p. injection of urethane (1.55 g kg^−1^). Following surgery (described below) neurophysiological recordings spanned the middle portion of the projected day (ZT 3–9). At the end of the experiment, mice received an overdose of urethane (2 g kg^−1^, i.p.) followed by transcardial perfusion.

### *In vivo* neurophysiology

Mice were prepared for stereotaxic surgery and insertion of multielectrode arrays as described previously (Howarth *et al*. 2014). In brief, urethane (1.55 g kg^−1^)‐anaesthetised mice were mounted in a stereotaxic frame (SR‐15M; Narishige International Ltd, London, UK). Body temperature was maintained at ∼37°C throughout all procedures via a homeothermic heat mat (Harvard Apparatus, Edenbridge, UK). Pupils were dilated by topical application of 1% (w/v) atropine solution and mineral oil was applied to retain corneal moisture (both Sigma‐Aldrich, Dorset, UK). The scalp was exposed by midline incision and, depending on the experiment, one or two craniotomies (∼800 μm diameter) drilled, centred on the stereotaxic coordinates specified below. Subsequently, recording probes (A4x8‐5mm‐50‐200‐177; Neuronexus, MI, USA) comprising four shanks (spaced 200 μm), each with eight recording sites (spaced 50 μm), were coated with fluorescent dye (CM‐DiI; Invitrogen, Paisley, UK) and inserted into the IGL/vLGN region (2.5 mm caudal and 2.2 mm medial to bregma) at a depth of 3.1 mm relative to the brain surface. In some experiments we placed a second electrode into the contralateral LGN to provide electrical stimulation or drug infusion (see below).

After allowing 30 min for neural activity to stabilise, wideband neural signals were acquired using a Recorder64 system (Plexon, Dallas, TX, USA), amplified (×2000) and digitised at 40 kHz. Action potentials were discriminated from these signals offline as ‘virtual’‐tetrode waveforms using custom MATLAB scripts (The MathWorks, Natick, MA, USA) as described previously (Howarth *et al*. 2014) and resulting waveforms were sorted manually using commercial principle components based software (Offline sorter, Plexon). Single unit isolation was confirmed by reference to MANOVA *F* statistics, J3 and Davies–Bouldin validity metrics (Offline sorter) and the presence of a distinct refractory period (>1.5 ms) in the interspike interval distribution.

**Figure 1:**
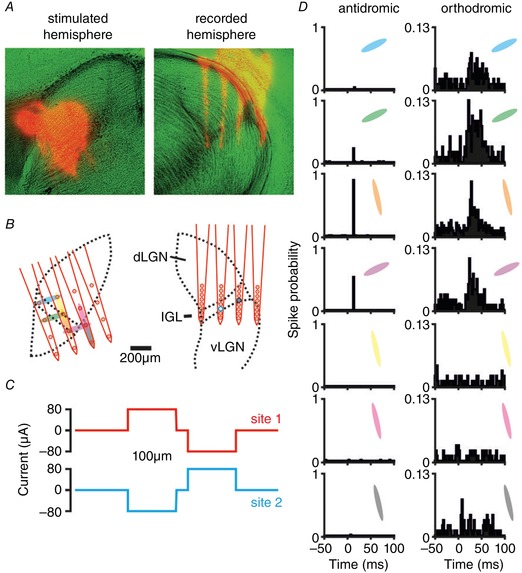
Identification of commissurally connected geniculate neurons *A*, anatomical images showing DiI‐labelled probe tracks (red) and light microscopy (pseudocoloured green). *B*, schematic representation of reconstructed stimulus (left) and recording (right) site locations relative to the LGN boundaries. In this example the stimulating probe was placed ∼250 μm rostral relative to the recording probe. Shaded ovals (left) indicate pairs of electrode sites used for current delivery and larger circles (right) indicate locations of two isolated neurons shown in panel *D*. *C*, current injection pattern used for these experiments; horizontally or vertically neighbouring pairs of sites were used to deliver biphasic dipolar current injection (in this case ±80 μA). *D*, perievent histograms showing response to electrical stimulation (100 trials) at the sites, and for the units, indicated in *B*. Visual responses of the orthodromically activated cell (right panel) are shown in Fig. 2 *F* (middle panel).

For electrical microstimulation experiments, a 16‐channel array (A4x4‐4mm‐200‐200‐1025; Neuronexus) was inserted into the contralateral IGL/vLGN region at an angle of 18° from vertical. Prior to probe insertion, electrode sites were coated in iridium oxide to increase charge carrying capacity (niPOD, Neuronexus). For stimulation, biphasic dipolar current pulses (100 μs/phase 70–120 μA) were delivered via selected pairs of electrode sites (Fig. 1 *B* and *C*; PlexStim; Plexon). Back voltage/currents were monitored continuously throughout the experiment to ensure effective stimulus delivery.

Contralateral thalamic inactivation was performed as described previously (Hanna *et al*. 2017). In brief, we implanted a 16‐site linear recording array (E16‐20mm‐100‐177; Neuronexus) attached to a drug cannula (outer diameter 165 μm) protruding 100 μm ventral to the probe tip into the IGL/vLGN region. The cannula underlying the recording probe was connected via flexible narrow bore tubing to a syringe pump (Harvard Apparatus, Cambridge, UK) preloaded with muscimol (1 mM in 0.9% saline; Sigma‐Aldrich) or vehicle (0.9% saline, pH 6.6). In these experiments, after evaluating baseline visual responses we infused muscimol or vehicle into the thalamus (2 μL at 1 μL min^−1^) while monitoring responses to visual stimuli (5 s binocular light steps 15.4 log photons cm^−2^ s^−1^). Drug onset was easily identifiable by a rapid loss of spontaneous and evoked neural activity, alongside a small increase in electrical noise.

### Visual stimuli

Light measurements were performed using a calibrated spectroradiometer (Bentham instruments, Reading, UK) and all visual stimuli were generated as described previously (Howarth *et al*. 2014; Allen *et al*. 2016). In brief, full‐field stimuli were generated via two LEDs (λ_max_ 410 nm; half‐width: ±7 nm; Thorlabs, Newton, NJ, USA), with intensity controlled via pulse width modulation using LabVIEW (National Instruments, Austin, TX, USA) and neutral density filter wheels (Thorlabs). Light was supplied to the subject via 7 mm diameter flexible fibre optic light guides (Edmund Optics, York, UK), positioned 5 mm from each eye and enclosed within internally reflective plastic cones to prevent any off‐target effects due to scattered light. At the chosen wavelength, all mouse photoreceptors display similar sensitivity such that, after correction for pre‐receptoral filtering, effective photon fluxes for the unattenuated stimuli differed by no more than 0.2 log units across photoreceptor classes (15.3–15.5 log photons cm^−2^ s^−1^; values reported below reflect effective irradiance for rod opsin, which was intermediate within this range).

Mice were maintained in darkness and 5 s light steps were applied in an interleaved fashion to contra‐ and/or ipsilateral eyes for a total of 10 repeats at logarithmically increasing intensities spanning 9.4–15.4 log photons cm^−2^ s^−1^ (interstimulus interval 20–50 s depending on intensity). For assessment of responses under light‐adapted conditions, we used the same apparatus and modulated LED intensity independently at either eye between 13.4 and 15.4 log photons cm^−2^ s^−1^ in pseudorandom sequence every 5 s (for a total of 20 repeats at each possible contrast/irradiance combination as illustrated in Fig. 6 *A*).

For receptive field (RF) mapping, stimuli were delivered via an LCD display (width: 26.8 cm; height: 47.4 cm; Hanns‐G HE225DPB; Taipei, Taiwan) angled at 45° from vertical and placed at a distance of ∼21 cm (occupying ∼63 × 96° visual angle), either directly in front of the animal or in some experiments rotated by 90° and positioned laterally at an angle of 30° relative to the animal's midline (in either the ipsilateral or the contralateral visual field). For eye‐specific stimulation, one LED was placed at the unstimulated eye and held at an equivalent irradiance to the background irradiance in the experimental room (∼14 log photons cm^−2^ s^−1^).

Stimuli were generated and controlled via MATLAB using the Psychophysics toolbox (Brainard, 1997; Pelli, 1997) and comprised white or black flashing bars (430 and 3.3 scotopic cd m^−2^ respectively, occupying ∼7° visual angle) superimposed on a background of the opposite polarity. Vertical or horizontal bars appeared at random locations (∼1.5° increments covering the display) for 250 ms followed by a blank screen for 250 ms (8 repeats per orientation/polarity/screen location). Stimulus blocks involving white or black bars were tested sequentially for each monitor location and viewing condition (i.e. ipsi‐/contralateral eye only and binocular viewing).

### Histology

At the end of the experiments, mice were perfused transcardially and the brain removed and sectioned (100 μm) and then mounted directly onto slides using Vectashield (Vector Laboratories Ltd.; Peterbourough, UK). DiI‐labelled probe placements were then visualised under a fluorescence microscope (Olympus BX51; Olympus UK, Southend‐on‐Sea, UK) with appropriate filter sets. Resulting images were then scaled to account for shrinkage (based on the known distance between electrode shanks) and aligned with appropriate stereotaxic atlas figures (Paxinos & Franklin 2001) using the optic tract, LGN and hippocampus as landmarks. Anatomical locations of recorded cells were estimated from these images, based on the projected location of the recording site where we observed the largest spike amplitude for that cell.

### Data analysis

#### Electrical stimulation

**Figure 2:**
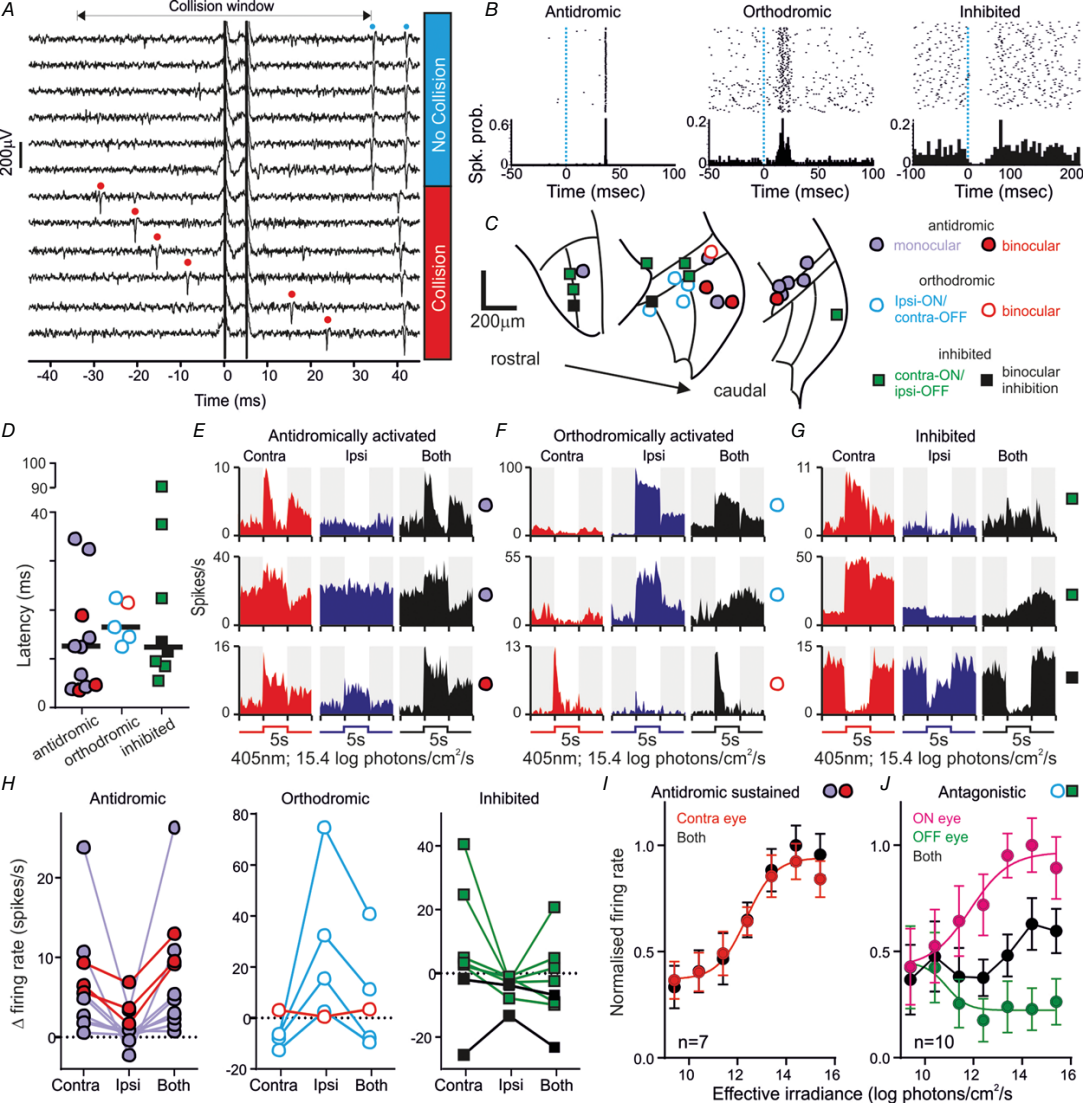
Properties of commissurally connected IGL/vLGN neurons *A*, example neuron antidromically activated from the contralateral IGL/vLGN (100 μA paired stimuli; 5 ms interstimulus interval) demonstrating abolition of the first evoked by spontaneous discharge (red dots) occurring within the collision window. *B*, rasters and histograms for antidromic, orthodromically activated or inhibited units following low frequency (0.5 Hz) electrical stimulation of the contralateral IGL/vLGN. *C*, anatomical locations of units based on connection type and visual response characteristics (key applies also to panels *D–H*). *D*, stimulus‐evoked response latencies for antidromic (*n* = 11), orhthodromic (*n* = 5) and inhibited cells (*n* = 8). *E–G*, histograms of response to monocular or binocular bright light steps for example antidromic (*E*), orthodromic (*F*) or inhibited cells (*G*). *H*, light‐evoked changes in firing rate for cells the across identified unit classes. *I*, normalised mean ± SEM firing rate during light stimulation from 7 antidromically activated units with sustained visual responses. Ipsilateral responses were omitted since only a subset (*n* = 3) responded. *J*, irradiance response relationship as in *I* for orthodromically activated (*n* = 4) or inhibited (*n* = 6) cells with antagonistic responses (monocular responses grouped according to whether the visual response was ON or OFF).

For analysis of responses to electrical stimulation we constructed histograms of spike counts relative to the time of stimulus onset (1 ms bin size smoothed with a 5 ms moving window; 100 trials). Cells were considered responsive when spike counts in one or more bins within 100 ms of the time of stimulus onset exceed the 99% confidence limits of the spike counts in the 200 ms epoch prior to stimulation. Latency measures reported here represent the timing of the first post‐stimulus bin where spike counts exceeded these limits. For cells exhibiting significant increases in firing, we further distinguished between orthodromic and antidromic activations based on previously established criteria (Zhang & Rusak, 1989; Blasiak & Lewandowski, 2013). Specifically, to be classed as antidromically activated the cell had to reliably fire evoked spikes at stable latency, follow high stimulation frequencies (200 Hz) and evoked spikes had to be abolished when the cell in question fired a spontaneous action potential just before or after electrical stimulation (collision test; Fig. 2 *A*).

#### Classification of visual response properties

Classification of visual response properties was based on responses of each cell to light steps applied from darkness to either eye independently or in combination over varying intensity (as described above). Responses were considered significant when the spike count in the 500 ms epochs immediately following the onset or offset of the stimulus (from 10 trials) exceed the 95% confidence limits of the pre‐stimulus firing activity (5 s epoch before stimulus onset). To be classified as visually responsive, a cell had to exhibit significant responses to at least two of the intensities tested. Cells were classed as exhibiting binocular excitation or inhibition when they exhibited significant responses of the same sign (i.e. increases or decrease in firing rate) when stimuli were applied to both contralateral and ipsilateral eyes independently. We additionally classified cells as showing antagonistic binocular responses when one or both of the following conditions were met: (1) we detected significant increases in firing driven by monocular stimuli presented to one eye and decreases in firing driven by the monocular stimulation of the other eye or (2) the response to binocular stimuli was significantly smaller than that for the most robust of the two monocular stimuli (Student's unpaired *t* test). Otherwise cells were classified as monocular.

#### Responses to thalamic inactivation

**Figure 3:**
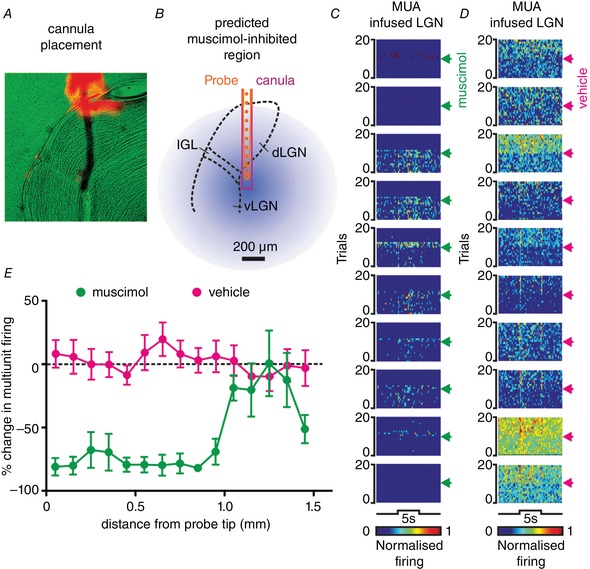
Validation of thalamic inactivation approach *A*, anatomical image showing DiI‐labelled probe track (red) and light microscopy (pseudocoloured green) for a drug‐probe placement within the LGN. *B*, corresponding schematic representation of recording probe (10 ventral‐most sites of the 16 channel probe), drug cannula and predicted spread of muscimol (based on data in *E* and assuming symmetrical spread of drug from probe tip). *C* and *D*, pseudocoloured rasters showing normalised spike counts for 10 ventral‐most sites across 20 trials of a bright binocular light step (405 nm LED, 5 s, 15.4 log effective photons cm^−2^ s^−1^) during which muscimol (*C*) or vehicle (*D*) was infused (2 μL at 1 μL min^−1^; timing indicated by arrowheads). *E*, mean ± SEM percentage change in multiunit activity (MUA) during the binocular light step following muscimol or vehicle infusion as a function of distance of the recording site from the probe tip (*n* = 7 muscimol and *n* = 8 vehicle experiments).

To validate the impact of drug infusion on the inactivated LGN (Fig. 3), for each electrode site on the drug‐probe we calculated the change in multiunit firing following muscimol or vehicle during the 5 s epochs in which bright light steps (15.4 log photons cm^−2^ s^−1^) were applied to both eyes (means of 10 trials pre‐ and post‐infusion). Since the electrode sites were a fixed distance from the tip of the drug infusion cannula, we then calculated the average change in multiunit firing across all experiments from the relevant groups as a function of distance from the probe tip using a two‐electrode site moving average. Sites with zero pre‐infusion firing were excluded from this average.

**Figure 4:**
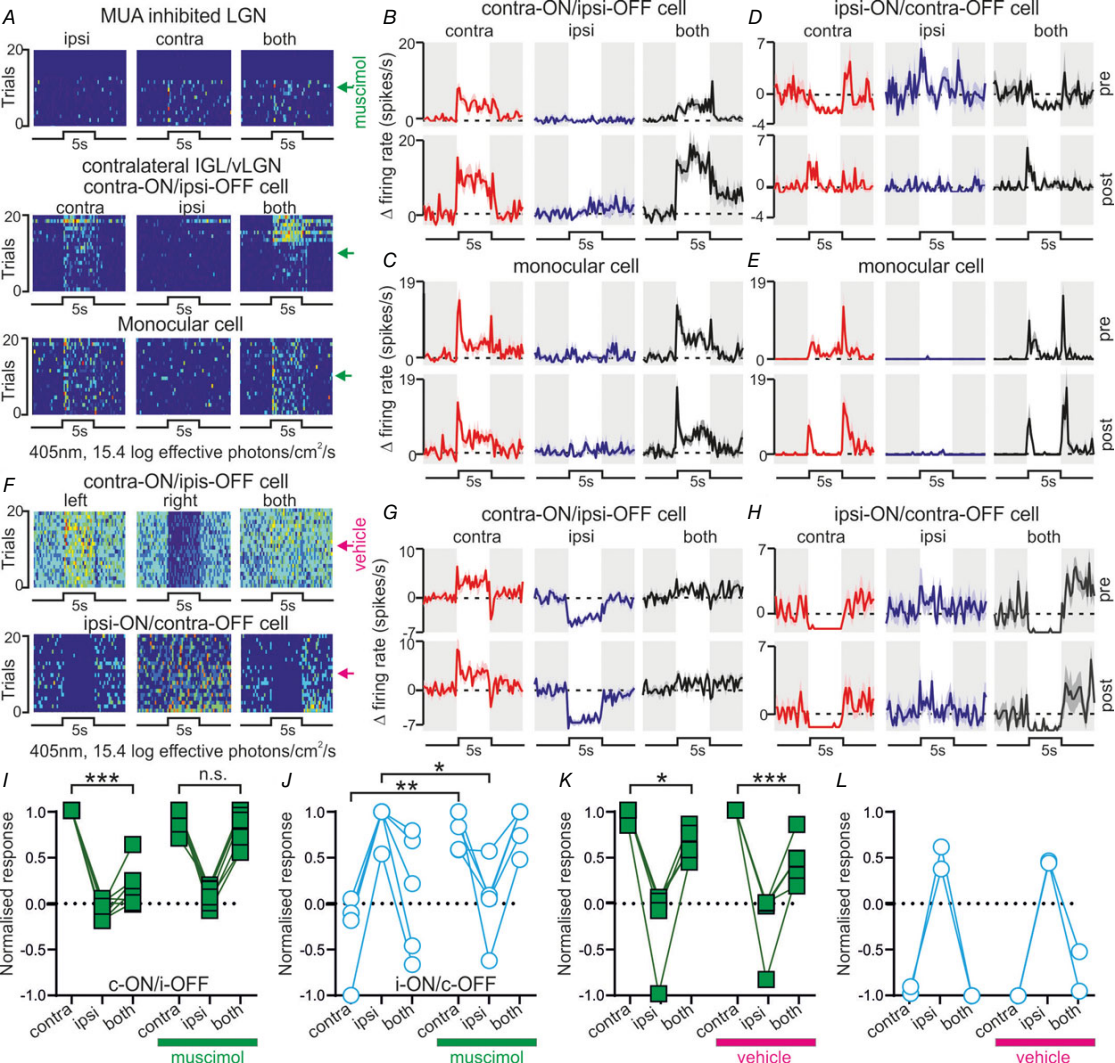
Thalamic inactivation modulates antagonistic binocular responses *A*, pseudocoloured spike count rasters across 20 trials in which bright light steps were applied to left, right or both eyes. Top panel: multiunit data from the LGN receiving muscimol infusion. Lower panels: isolated units from the contralateral IGL/vLGN. Arrows denote timing of muscimol infusion. *B* and *C*, mean ± SEM change in firing for individual cells before or after muscimol infusion into the contralateral LGN (upper and lower panels, respectively); contra‐ON/ipsi‐OFF (*B*) and monocular cell (*C*) correspond to rasters shown in middle and lower panels of *A*, respectively. *D* and *E*, responses of an ipsi‐ON/contra‐OFF (*D*) and monocular cell (*E*) from a second experiment (conventions as above). *F*, rasters as in *A* for antagonistic IGL/vLGN cells following infusion of vehicle (saline) into the contralateral LGN. *G* and *H*, mean ± SEM change in firing for cells shown in *F* before or after vehicle infusion into the contralateral LGN. *I–L*, normalised response for individual contra‐ON/ipsi‐OFF (*I* and *K*) and ipsi‐ON/contra‐OFF (*J* and *L*) cells before and after muscimol (*I* and *J*) or vehicle infusion (*K* and *L*) into the contralateral LGN. Data were analysed by two‐way RM‐ANOVA (Table 2) with Sidak's *post hoc* test; n.s., *P* > 0.05; ^*^ *P* < 0.05, ^**^ *P* < 0.01, ^***^ *P* < 0.001.

To quantify changes in sensory properties following thalamic inactivation (Fig. 4) we then calculated the mean response of each neuron (classified based on pre‐infusion visual responses as above) to bright light steps targeting the ipsi‐/contralateral or both eyes (means of 10 trials pre‐ and post‐infusion). To remove generalised changes in cell responsiveness as a source of variability, response amplitudes for each cell were normalised according to the largest absolute magnitude response separately for pre‐ and post‐infusion. Data from each group of visually responsive neurons was then analysed by two‐way repeated measures (RM) ANOVA with Sidak's *post hoc* test.

#### Visual responses under light adapted conditions

For analysis of data where full field modulations were applied under light adapted conditions (Figs. 6 and 7), within each 5 s block of the stimulus there were four possible combinations of ipsi‐ and contralateral irradiance, each of which could be preceded by any one of those four combinations (giving a total of 16 possible stimuli). We then first calculated the mean firing rate over time for each of these 16 possible stimuli (250 ms bins size; 20 trials each). The resulting data were then normalised by subtracting the global mean (across all stimuli/time points) and then dividing by the largest absolute deviation from that value observed in any of the 16 time series. Of the 16 time series, there were four conditions (corresponding to each of the four possible binocular irradiance combinations) in which stimulus intensity remained unchanged relative to the previous 5 s. We took the average firing rate across each of these four time series as an estimate of the contrast‐independent components of the cells’ responses to that binocular irradiance. To estimate contrast‐dependent influences on the cells’ activity we then took data from the remaining 12 time series (presented in Figs. 6 *C* and 7 *B*) and subtracted the contrast‐independent component for the relevant binocular irradiance. The contrast component for each stimulus type (i.e. increase, decrease or no change for ipsi‐ and/or contralateral intensity) was then quantified as the largest absolute magnitude deviation from the above within 1 s of stimulus onset. Where there was more than one stimulus block reflecting the same type of stimulus (i.e. for contra‐ or ipsilateral‐only stimuli) we took the average of the two different conditions representing that type of contrast. Data for contrast‐dependent and ‐independent responses for each class of visually responsive neuron were than analysed by one‐way RM‐ANOVA with one‐sample Student's *t* test or Sidak's *post hoc* test, respectively.

#### Receptive field mapping

RF parameters (Figs. 8 and 9) were determined separately for vertical and horizontal bars for each screen position and/or eye(s) viewing the stimulus by calculating (for each bar position) the mean change in firing following the appearance of white bars minus the mean response following the appearance of black bars (100 ms epochs starting 35–125 ms after bar appearance). The resulting profiles were then fit with 1‐D Gaussians to estimate receptive field centre position and diameter (full width at half‐maximum). Values for diameter reported here represent the averages of estimates obtained using vertical and horizontal bars corrected according to visual angle. For calculations of visual angle, tangent correction was applied in the azimuthal direction only.

## Results

### Visual inputs to commissurally connected IGL/vLGN neurons

We first aimed to understand the basic visual response properties of cells projecting to or receiving input from the contralateral visual thalamus in mice. To this end, we implanted 32‐channel multielectrode recording probes targeting the IGL/vLGN region and 16‐channel stimulating probes into the contralateral LGN (Fig. 1 *A* and *B*). We then delivered dipolar current pulses across pairs of electrode sites and used established electrophysiological criteria (see methods) to identify antidromic and orthodromically responding cells (Fig. 1 *C* and *D*).

In total we performed multielectrode extracellular recordings in 15 mice, from which we were able to isolate 208 individual neurons across the IGL/vLGN or ventral portions of the dLGN (*n* = 158 and 50 respectively). Table 1 shows the classification of neurons making up this sample, according to their basic visual response properties and their responses to electrical stimulation of the contralateral thalamus (see ‘Data Analysis’ section of the Methods for further details of classification). For these experiments, we opted to deliver relatively modest current via our stimulating electrode (≤120 μA) so as to limit the activation of neurons outside of the LGN region. As a consequence, we did not expect our stimuli to activate neurons across the full rostro‐caudal extend of the IGL/vLGN (which spans ∼1 mm). We therefore draw no inferences as to the commissural connectivity (or lack thereof) for cells that did not exhibit detectable response to electrical stimulation.

**Table 1:** Classification of identified neurons based on visual response properties and response to stimulation of the contralateral visual thalamus

	Antidromic		Orthodromic		Inhibited		None	Total
Visual response	*n*	*P*	*n*	*P*	*n*	*P*	*n*	*n*
Monocular	8	0.751	0	0.006	0	0.0003	127	135
Binocular	3	0.096	1	0.408	0	∼1	16	20
Contra‐ON/ipsi‐OFF	0	∼1	0	∼1	6	0.0001	5	11
Ipsi‐ON/contra‐OFF	0	∼1	4	0.0002	0	∼1	11	15
Binocular Inhibited	0	∼1	0	∼1	2	0.038	5	7
No response	0	0.605	0	∼1	0	∼1	20	20
Total cells	11		5		8		184	208

Table shows the number of cells of each class identified in contralateral LGN stimulation studies. Within each class of commissurally connected cells, the proportions of each visual response type were compared with those in the total sample of cells by Fisher's exact test and the *P*‐values represent corresponding probabilities.

Across the 15 experiments we performed, we identified 11 cells that passed the criteria for antidromic activation (Fig. 2 *A* and *B*), all of which were located within or bordering the IGL/vLGN region (Fig. 2 *C*). Based on the location(s) from which we were able to evoke antidromic spikes (Fig. 1) we determined that these cells similarly projected to the contralateral IGL/vLGN or close thereby. We also identified five cells in the IGL/vLGN region which were excited by stimulation of the contralateral LGN but did not meet the criteria for antidromic activation (orthodromically activated) and a further eight cells that were inhibited following contralateral stimulation (Fig.  2 *B* and *C*). The latency of stimulus‐evoked changes in firing across the three groups of cells was statistically equivalent (Fig. 2 *D*; median ± SD: 12.1 ± 11.0, 16.5 ± 4.4 and 12.5 ± 28.4 ms, respectively; Kruskal–Wallis test, *P* = 0.08). It was noteworthy, however, that we observed considerable variability in response latencies, presumably reflecting stimulation of axonal pathways with varying fibre diameter, myelination and/or trajectory (Moore *et al*. 2000). In any case, for antidromic cells, the range of latencies observed here (3.8–34.3 ms) was very similar to those reported previously (Zhang & Rusak, 1989; Blasiak & Lewandowski, 2013). We also noted that, as expected, orthodromically activated cells were readily distinguished from antidromically activated cells by a significantly greater trial to trial variation (∼100‐fold) in the latency of evoked spikes (mean ± SEM jitter = 0.14 ± 0.03 *vs*. 11.13 ± 3.23 ms, respectively; *t* test, *P* < 0.001).

To determine the response of these various groups of commissurally connected cells to visual stimulation, we next applied full‐field light steps (405 nm LED; 5 s duration) across a wide range of light intensities (9.4–15.4 log effective photons cm^−2^ s^−1^) to the contralateral, ipsilateral or both eyes simultaneously. Of note, the population of cells identified as antidromically activated reliably increased firing rate in response to light increments applied to the contralateral eye and exhibited no response (*n* = 8/11; ‘monocular’ neurons) or more modest increases in firing (*n* = 3; ‘binocular’ neurons) in response to stimulation of the ipsilateral eye (Fig. 2 *E* and *H*; Table 1). Visually evoked responses in this group of cells consistently became apparent at low intensities (9.4–10.4 log effective photons cm^−2^ s^−1^) and in the majority of cases (*n* = 7/11) were maintained throughout light application (Fig. 2 *E*) such that steady state firing rates increased as a function of irradiance across the range typically encountered around twilight (Fig. 2 *I*). In summary, these data suggest that, in common with many other IGL/vLGN neurons (Harrington & Rusak, 1989; Harrington, 1997; Thankachan & Rusak, 2005; Howarth *et al*. 2014), the primary source of visual information conveyed by commissurally projecting IGL/vLGN cells is irradiance detected by the contralateral eye.

Unlike the antidromically activated cells, neurons exhibiting either orthodromic activations or inhibitions always displayed evidence of some form of binocular response (Table 1; Fig. 2 *F*, *G* and *H*). These binocular influences were usually characterised by opposing (antagonistic) responses to stimulation of the ipsi‐ and contralateral eyes. Moreover, across both orthodromically activated and inhibited cells, the sign of the response to electrical stimulation of the contralateral thalamus (excitatory or inhibitory) always matched that of their response to ipsilateral visual stimuli. Hence, all five orthodromically activated cells exhibited excitatory/ON responses driven by ipsilateral visual stimuli and four of these also exhibited inhibitory/OFF responses to contralateral visual stimuli (‘ipsi‐ON/contra‐OFF’; Fig. 2 *F* and *H*). The remaining cell in this case exhibited a strong excitatory contralateral visual response (classified here as ‘binocular’).

Similarly all eight cells that were inhibited by electrical stimulation of the contralateral LGN exhibited inhibitory/OFF responses to ipsilateral visual stimuli and six of these also displayed excitatory/ON responses to contralateral visual stimuli (‘contra‐ON/ipsi‐OFF’; Fig. 2 *G* and *H*). Here the remaining two cells exhibited inhibitory contralateral visual responses.

In sum, then, while we observe some variation in the visual response properties of cells that receive excitatory or inhibitory input from the contralateral LGN, a substantial proportion of these exhibit antagonistic binocular responses. We previously identified cells with this type of response in the IGL/vLGN (Howarth *et al*. 2014). In line with our earlier data, we here found that both populations of antagonistic cells reliably exhibited sustained responses to visual input such that their firing rates systematically varied in magnitude for monocular stimuli between 10.4 and 13.4 log photons cm^−2^ s^−1^ but changed relatively little following binocular stimulation (Fig. 2 *J*).

Of note, our previous work also indicated that cells with antagonistic binocular responses were essentially absent in the dLGN and rare within the IGL/vLGN (collectively <20% of cells; Howarth *et al*. 2014). Consistent with those data, across the full sample of LGN neurons recorded as part of this study (including those that did not respond to electrical stimulation of the contralateral thalamus), we found that antagonistic cells were similarly rare (*n* = 15/208 ipsi‐ON/contra‐OFF and 11/208 contra‐ON/ipsi‐OFF). By contrast, the proportions of antagonistic cells among those we identified as orthodromically activated (*n* = 4/5 ipsi‐ON/contra‐OFF) or inhibited (*n* = 6/8 contra‐ON/ipsi‐OFF) were significantly higher (Table 1; Fisher's exact text, *P* = 0.0002 and *P* < 0.0001 respectively).

These observations, therefore, support the view that antagonistic binocular responses are a specific feature of cells that receive commissural input, rather than simply a general feature of IGL/vLGN visual input. Indeed, a sizeable fraction of all the cells exhibiting antagonistic responses (4/15 ipsi‐ON/contra‐OFF and 6/11 contra‐On/ipsi‐OFF) we detected were also experimentally confirmed to receive input from the contralateral LGN.

### Contribution of commissural signalling to IGL/vLGN visual response properties

Given our data above, we speculated that commissural projections were likely pivotal in generating the antagonistic eye‐specific responses identified in the IGL/vLGN of mice and hamsters (Harrington & Rusak, 1989; Howarth *et al*. 2014). Specifically we hypothesised that response components driven by ipsilateral retinal stimulation may be dependent on commissural input. To test this inference, we next evaluated visual responses across the IGL/vLGN region (as described above) before and after inactivation of the contralateral visual thalamus by local infusion of the GABA_A_ agonist muscimol.

To this end, we implanted a recording probe with attached drug cannula (preloaded with 1 mM muscimol) into the contralateral LGN, allowing us to monitor spontaneous/light‐evoked neural activity and confirm its abolition following muscimol infusion (Fig. 3 *A–C*). As we have reported previously (Hanna *et al*. 2017), 2 μL muscimol infusions resulted in robust and widespread inhibition of neural activity (Fig. 3 *E*; experiments performed in 7 mice) across a region predicted to encompass the vast majority of the contralateral LGN (assuming a uniform diffusion of drug from the injection site). By contrast, neural activity was robustly maintained in equivalent experiments (*n* = 8) in which we instead performed vehicle infusions (Fig. 3 *D* and *E*).

We next evaluated the influence of contralateral thalamic inactivation on visually evoked responses in the IGL/vLGN. From seven mice where we performed contralateral muscimol infusions we were able to isolate 122 visually responsive neurons of which 12 exhibited antagonistic visual responses prior to muscimol infusion. This included seven cells with contra‐ON/ipsi‐OFF responses (Fig. 4 *A*, *B* and *I*) and five cells with ipsi‐ON/contra‐OFF responses (Fig. 4 *D* and *J*). Importantly, for both types of cells, two‐way RM‐ANOVA revealed a significant change in the relative magnitude of responses to bright 5 s light steps targeting the contralateral, ipsilateral or both eyes following muscimol infusion (visual stimulus × treatment interactions; Table 2).

**Table 2:** Effect of contralateral thalamic muscimol or vehicle infusions on visual response properties

			Two‐way RM‐ANOVA probability		
Visual response	Treatment type	No. of cells	Stimulus	Treatment	Stimulus × treatment
Contra‐ON/ipsi‐OFF	Muscimol	7	<0.0001	0.0012	0.0002
	Vehicle	5	<0.0001	0.3268	0.1009
Ipsi‐ON/contra‐OFF	Muscimol	5	0.2214	0.2330	0.0002
	Vehicle	2	NA	NA	NA
Monocular	Muscimol	102	<0.0001	0.7216	0.9083
	Vehicle	63	<0.0001	0.1574	0.5859
Binocular	Muscimol	8	0.2471	0.2390	0.3907
	Vehicle	13	0.0002	0.0772	0.0575

Result of two‐way RM‐ANOVA analysis of normalised visual response amplitudes for each class of IGL/vLGN neuron following either muscimol or vehicle infusion into the contralateral visual thalamus. The effect of vehicle treatment on Ipsi‐ON/Contra‐OFF cells was not analysed due to the small sample size. NA, not applicable.

In the case of contra‐ON/ipsi‐OFF cells, which had relatively low baseline (dark‐adapted) firing rates (mean ± SEM: 1.3 ± 0.3 spikes s^−1^), the antagonistic influence of the ipsilateral eye was most evident as a significantly reduced light‐evoked firing in response to binocular *vs*. contralateral‐only light steps (Fig. 4 *I*, Sidak's *post hoc* test, *P* < 0.0001). Following infusion of muscimol into the contralateral visual thalamus this difference in response to binocular *vs*. contralateral‐only light steps disappeared (Fig. 4 *I*, Sidak's *post hoc* test, *P* = 0.92), converting the response of these cells into a simple ON excitation driven by the contralateral eye. Importantly, we did not observe any significant change in the sensory properties of five cells with contra‐ON/ipsi‐OFF responses that were identified in experiments in which we infused vehicle into the contralateral LGN (Fig. 4 *F*, *G* and *K*, Table 2). Indeed, for these five cells we found a significant reduction in the response binocular *vs*. contralateral‐only light steps both before and after vehicle infusion (Fig. 4 *K*, Sidak's *post hoc* test, *P* = 0.017 and *P* = 0.009 respectively).

The effects of thalamic inhibition on the ipsi‐ON/contra‐OFF cells were more complex. Thus, while we observed a reduction in the ipsilateral ON component of the cells’ response post‐muscimol infusion (Fig. 4 *J*; Sidak's *post hoc* test, *P* = 0.02), the contralateral‐OFF response also disappeared, leaving instead a transient ON excitation (Fig. 4 *J*, Sidak's *post hoc* test, *P* = 0.006). Thus, as above, responses of the ipsi‐ON/contra‐OFF cells were also converted to an ON excitation driven by the contralateral eye when we removed input from the contralateral LGN. Further, equivalent behaviour was not observed in two ipsi‐ON/contra‐OFF cells that we identified in experiments involving vehicle infusion into the contralateral LGN (Fig. 4 *F*, *H* and *L*). Instead both cells retained both contralateral‐OFF and ipsilateral‐ON components of their response.

During our thalamic inactivation experiments, in addition to antagonistic cells, we also identified much larger numbers of cells with monocular (contralateral‐driven) visual responses (Fig. 4 *A*, *C* and *E*; *n* = 102). Analysis with two‐way RM‐ANOVA did not reveal any significant change in the ocular response preferences of this group of cells, nor in the equivalent group of neurons (*n* = 63) identified in vehicle infusion experiments (Table 2). Similarly, the same analysis performed on neurons that exhibited excitatory responses to stimulation of both contralateral and ipsilateral eyes (‘binocular’ cells) did not reveal any significant change in responsiveness following muscimol or vehicle infusion (Table 2; *n* = 8 and *n* = 13 cells tested respectively). In summary, these data indicate that inactivation of the contralateral thalamus does not result in a global change in visual processing in the intact IGL/vLGN and instead seems to selectively impact the properties of cells with antagonistic binocular responses.

**Figure 5:**
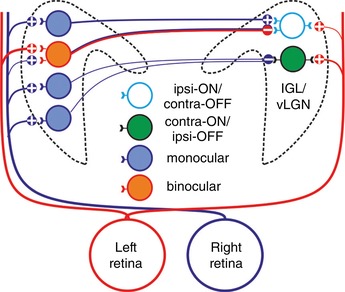
Proposed circuitry generating antagonistic eye‐specific responses Our data suggest that, for cells with contra‐ON/ipsi‐OFF antagonistic responses, contralateral ON responses are driven by retinal input while ipsilateral OFF responses are driven by inhibitory input from monocular (contralateral ON) cells in the opposite LGN. For ipsi‐ON/contra‐OFF cells, both response components involve commissural signalling. These cells receive excitatory commissural input (presumably via monocular cells that receive crossed retinal inputs) that drives ipsilateral ON responses. By contrast, their contralateral OFF responses are predicted to derive from commissurally projecting neurons that receive binocular retinal input biased towards uncrossed projections. In addition to a weak contralateral ON input from the retina, this arrangement is predicted to generate well balanced antagonistic inputs.

Collectively, our data confirm that antagonistic visual responses in the mouse IGL/vLGN region specifically rely on input from the contralateral hemisphere. In particular, we find that the ipsilateral responses of both classes of antagonistic cell are strongly reliant on commissural input. By contrast, commissural projections differentially contribute to antagonistic cell responses to contralateral light steps; ON responses persist following inactivation of the contralateral LGN while OFF responses do not. Alongside the data from our thalamic stimulation experiments reported previously in the article we therefore propose that contra‐ON/ipsi‐OFF and ipsi‐ON/contra‐OFF antagonistic responses are mediated by distinct neural circuits (Fig. 5; see Discussion for further explanation).

### Sensory properties of antagonistic IGL/vLGN neurons

Having established a key role for commissural input in generating the binocular responses of antagonistic cells, we next aimed to define the sensory properties of such cells in more detail. The data presented above and previously (Howarth *et al*. 2014) suggest that antagonistic cells may function to encode interocular differences in brightness. However, since the stimuli we have evaluated to date were applied under dark adapted conditions we have only been able to observe how such cells respond to light increments. Here we assessed antagonistic cell responses under light‐adapted conditions (mean irradiance = 14.4 log effective photons cm^−2^ s^−1^), where we applied 2‐log unit increments and decrements to either eye independently in pseudorandom order (5 s interstimulus interval). As described below, this approach had the additional benefit in that it allowed us to dissociate components of the responses that were dependent on the irradiance presented to either eye *vs*. those that were specifically driven by the change in stimulus intensity (‘contrast’).

**Figure 6:**
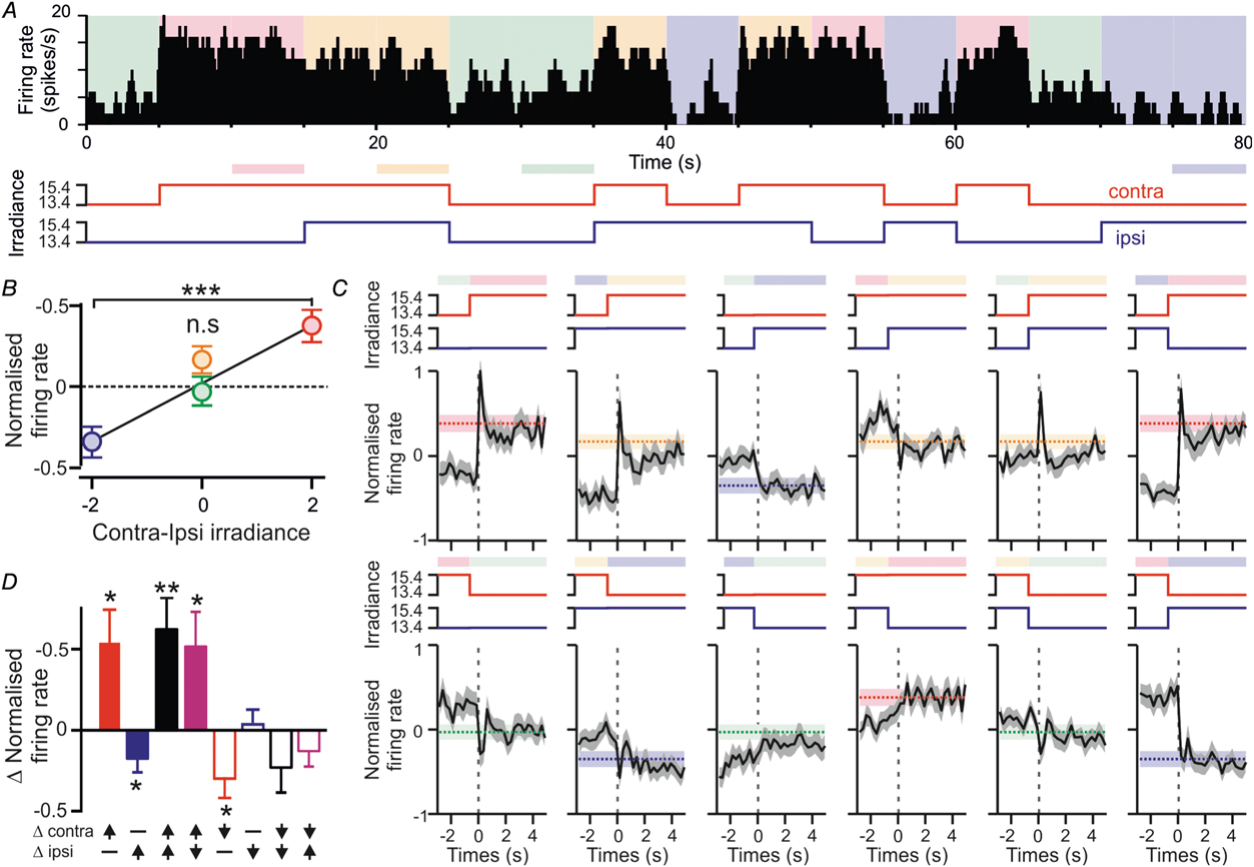
Contralateral‐ON antagonistic cell firing encodes interocular difference in irradiance *A*, firing rate of a contra‐ON/ipsi‐OFF cell across a segment of a randomised binocular luminance protocol, illustrating all possible stimulus transitions. Coloured shading represents the combination of ipsi‐ and contralateral irradiance at each point. Lower panel illustrates changes in irradiance at either eye; shaded bars represent 5 s stimulus blocks where binocular irradiance did not change relative to the previous 5 s. *B*, mean ± SEM normalised firing activity (relative to mean across whole protocol) for contra‐ON/ipsi‐OFF cells (*n* = 33) over each of the four stimulus blocks highlighted in *A* (lower panel) and plotted according the interocular difference in irradiance. *C*, mean ± SEM normalised firing activity for contra‐ON/ipsi‐OFF cells (as above) over time relative to a change in ipsi‐ and/or contralateral irradiance. Shaded bars represent the expected post‐step firing rate in the absence of visual contrast (mean ± SEM; re‐plotted from *B*). *D*, mean ± SEM isolated contrast responses (difference in firing relative to that expected for the irradiance) following changes in ipsi‐ and/or contralateral irradiance. Data in *B* and *D* analysed by one‐way RM‐ANOVA with Sidak's *post hoc* test (*B*) or one‐sample *t* test (*D*); n.s, *P* > 0.05; ^*^ *P* < 0.05, ^**^ *P* < 0.01, ^***^ *P* < 0.001.

The majority of the antagonistic cells recorded as part of this study were tested in this paradigm (*n* = 30/33 ipsi‐ON cells and *n* = 33/42 contra‐ON cells from 57 of the 67 experiments including those described above as well as additional experiments for analysis of spatial response properties below). An example of the response of one of these contra‐ON/ipsi‐OFF neurons during a small portion of this protocol is shown in Fig. 6 *A* to illustrate key features of the experimental design. Since each eye could experience one of two intensities, within each 5 s stimulus epoch there were four possible combinations of ipsi‐ and contralateral irradiance (each of which could in turn have been preceded by any of those 4 conditions). For each experiment, we first identified all those epochs in which the combination of ipsi‐ and contralateral irradiance remained unchanged relative to the preceding 5 s (as illustrated in Fig. 6 *A*). Since, by definition, responses dependent on visual contrast must rapidly adapt (to allow the cell to detect further changes in the stimulus), our expectation was that any such influence should be negligible by the start of these epochs. As such, we took the mean firing rate of each cell across the identified 5 s blocks as a measure of the contrast‐independent influence on that cells activity (Fig. 6 *B*).

Across the 33 contra‐ON/ipsi‐OFF cells tested in this paradigm we found a strong influence of binocular irradiance on firing rate (Fig. 6 *B*, one‐way RM‐ANOVA, *P* < 0.0001). Importantly here, firing rates were significantly higher under conditions of high contra‐ and low ipsilateral irradiance relative to the converse pattern of binocular irradiance (Sidak's *post hoc* test, *P* < 0.0001). By contrast, there was no significant variation in firing rate between conditions in which irradiance was uniformly high or low for both eyes (Sidak's *post hoc* test, *P* = 0.35). These data therefore indicate that, under light‐adapted conditions, the basal firing rate of contra‐ON/ipsi‐OFF cells reliably provides information about the interocular difference in irradiance.

We next evaluated how the firing rate of these contra‐ON/ipsi‐OFF cells immediately following a change in ipsi‐ and/or contralateral light intensity (which potentially contains information about stimulus irradiance and contrast) compared to that observed in the absence of prior contrast. Strikingly, while firing rates immediately following a step to any given binocular irradiance did diverge from the relevant contrast‐independent measures reported above, this effect was very transient (Fig. 6 *C*). Indeed, within 2 s of a change in light intensity at either eye (or both eyes), firing rates very closely matched those shown in Fig. 6 *B*, regardless of how that binocular irradiance combination was reached. This observation further supports our conclusion that such cells encode interocular differences in irradiance. Nonetheless, the transient divergence in firing rates shown in Fig. 6 *C* clearly also highlights components of the cell's responses that are not explained by this parameter. We extracted this contrast‐dependent component for each combination of ipsi‐ and/or contralateral light step by taking the peak firing rate following the change in light intensity and subtracting the expected (contrast‐independent) rate. The resulting contrast‐dependent responses provided evidence of significant contralateral‐ON and more modest ipsilateral‐OFF influences (Fig. 6 *D*). However, the contralateral‐ON signals were clearly dominant since the responses to simultaneous increases in light intensity presented to both eyes were similar to those observed following an increase in light intensity applied to the contralateral eye only (one‐way ANOVA with Sidak's *post hoc* test, *P* > 0.999).

**Figure 7:**
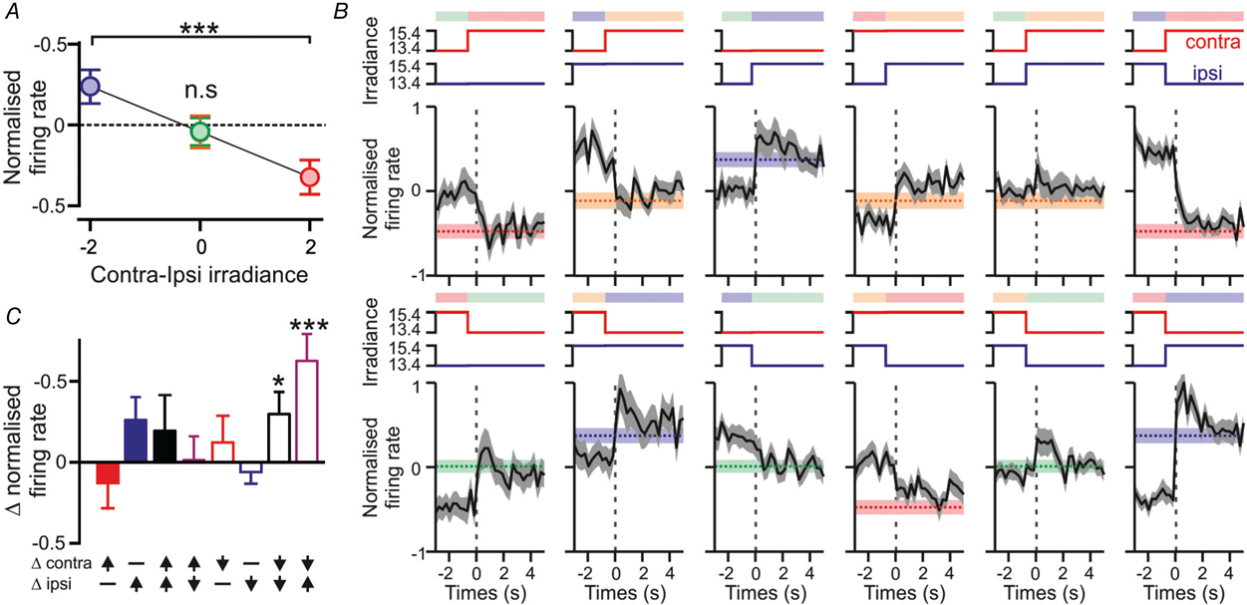
Interocular irradiance coding in ipsilateral‐ON antagonistic cells *A*, mean ± SEM normalised firing activity as a function of interocular difference in irradiance for ipsi‐ON/contra‐OFF cells (*n* = 30). *B*, mean ± SEM normalised firing activity for Ipsi‐ON/contra‐OFF cells (as above) over time relative to a change in ipsi‐ and/or contralateral irradiance. Shaded bars represent the expected post‐step firing rate in the absence of visual contrast (mean ± SEM; re‐plotted from *A*). *C*, mean ± SEM isolated contrast responses following changes in ipsi‐ and/or contralateral irradiance. Conventions and analysis throughout as for Fig. 6 *B–D*. n.s, *P* > 0.05; ^*^ *P* < 0.05, ^***^ *P* < 0.001.

We next applied a similar set of analyses to the responses of ipsi‐ON/contra‐OFF cells tested in this paradigm (Fig. 7; *n* = 30). Analogous to the situation described above, the basal firing rates of this group of antagonistic cells were strongly influenced by interocular differences in irradiance. Hence, ipsi‐ON/contra‐OFF cells exhibited significantly higher firing rates when there was low contra‐ and high ipsilateral irradiance, rather than the converse (Fig. 7 *A*; one‐way ANOVA with Sidak's *post hoc* test, *P* < 0.0001). Moreover, like contra‐ON‐ipsi‐OFF cells, this group of neurons exhibited essentially identical firing rates in the face of uniformly high or low irradiance at both eyes (Sidak's *post hoc* test, *P* = 0.594). This relationship between firing rate and interocular irradiance difference was similarly strongly maintained following acute changes in light intensity at ipsi‐ and/or contralateral eyes (Fig. 7 *B*). Indeed, the isolated contrast‐dependent response components of these cells were generally less reliable than those of their contra‐ON/ipsi‐OFF counterparts (Fig. 7 *C*); while there was a trend towards increased firing rates for ipsilateral ON and contralateral OFF contrast, the most pronounced changes were observed when both of these events occurred simultaneously (i.e. a decrease in contralateral irradiance and an increase in ipsilateral irradiance; one‐sample *t* test, *P* = 0.0007).

Collectively, our data above demonstrate that antagonistic cells uniquely encode information about interocular differences in irradiance, under diffuse illumination. Indeed, equivalent analysis to that shown in Figs. 6 and 7 did not reveal an equivalent effect across any of the IGL/vLGN cells with monocular or more conventional binocular responses recorded in the same experiments (*n* = 267 and *n* = 45 respectively; not shown).

A significant remaining question, however, is the extent to which the properties described above rely on the spatial distribution of light incident on each retina. The stimuli we have applied so far have uniformly modulated light intensity for each eye, but it is possible that the resulting responses were in fact dependent on the radiance within a particular region (or regions) of visual space. The presence of such discrete receptive fields (RFs) underlying the antagonistic cell responses potentially has significant implications for the nature of the visual signal those cells encode. For example, since the IGL/vLGN has been implicated in visuomotor control, one possibility is that ipsi‐ and contralateral inputs to the antagonistic cells arise from portions of the retina covering discrete, overlapping regions of visual space such that these cells change their firing as a function of binocular convergence. To investigate this, we therefore set out to determine the size and location of antagonistic RFs.

**Figure 8:**
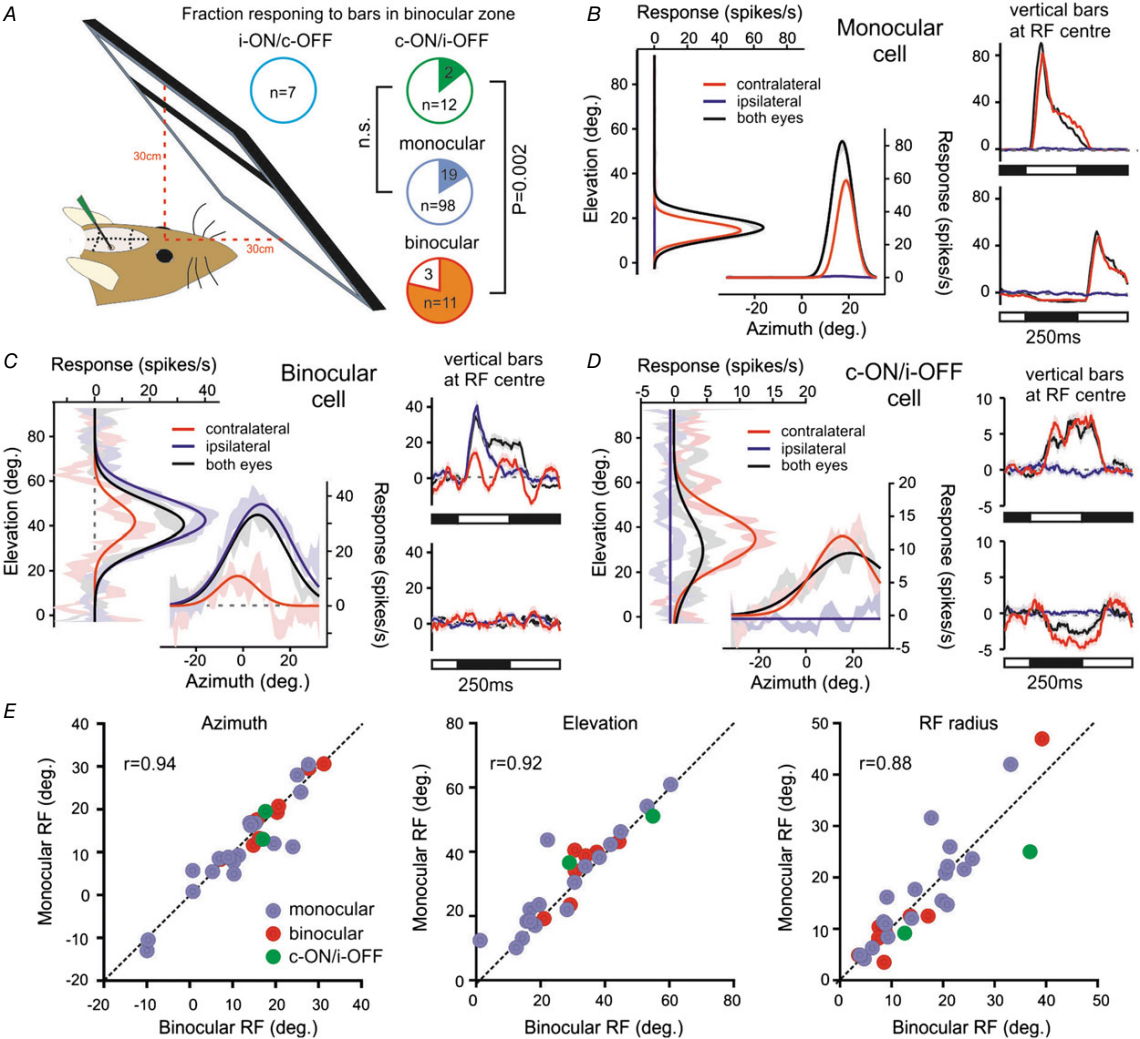
Antagonistic cell receptive fields do not preferentially localise to binocular visual space *A*, schematic representation of visual display placement (occupying ∼70% of binocular visual space) and the proportion of each class of IGL/vLGN neurons responding to the appearance of horizontal and vertical bars (filled regions in pie charts). Data were analysed by Fisher's exact test; n.s., *P* > 0.05. *B–D*, responses of individual monocular (*B*), binocular (*C*), and contra‐ON/ipsi‐OFF antagonistic cells (*D*) to bar stimuli presented to one or both eyes. In all cases, left panels show mean ± SEM difference in response to white *vs*. black horizontal or vertical bars as a function of bar position (in degrees relative to midpoint between the eyes). Continuous lines show Gaussian fits used to estimate RF position and diameter. Right panels show mean ± SEM change in firing evoked by vertical white (top) or black (bottom) bars appearing at the RF centre (data for horizontal bars omitted for clarity). *E*, relationship between RF parameters obtained for cells that responded under both monocular *vs*. binocular viewing conditions (*n* = 2 contra‐ON/ipsi‐OFF, 18 monocular and 8 binocular cells).

Since only a relatively restricted region of the mouse visual field is actually visible to both eyes, we started by placing a visual display directly in front of the mouse (Fig. 8 *A*) so as to cover the majority of this binocular visual field (∼70%). We then applied flashing horizontal or vertical bars (occupying ∼7° visual angle) at varying positions and polarities separately for conditions when either the ipsi‐ or contralateral eye was occluded or when both eyes could view the stimulus (see Methods for full details). In total, we tested these stimuli in 24 mice, from which we isolated 202 IGL/vLGN neurons which included, based on their responses to full field light steps, 21 cells with antagonistic responses (*n* = 7 ipsi‐ON/contra‐OFF and *n* = 14 contra‐ON/ipsi‐OFF), 117 cells with monocular (contralateral driven responses) and 14 cells exhibiting binocular excitations.

Across the monocular cells (which made up the majority of our sample) we found only a small subset (*n* = 19/117; ∼16%) that responded to the appearance of bar stimuli. This likely reflects the fact that RFs for the remaining cells were located in regions of visual space not covered by our display. Indeed, in mice, the full visual field for each eye is very large (near 180°) and our visual display only occupied a small fraction of that region. In any case, an example of the responses from one of the monocular neurons where we were able to map an RF is shown in Fig. 8 *B*. There, we plot the difference in the firing rate response following the appearance of a white *vs*. black bars as a function of the position where the bar appeared. This reveals a discrete portion of the visual field where the appearance of light bars increases firing rate and dark bars decreases firing rate (as illustrated in the rightmost panels of Fig. 8 *B*). Similar responses were obtained when stimuli were presented to just the contralateral eye or both eyes, while no response was observed under the ipsilateral‐only viewing condition. The cell was therefore categorised as having a contralateral‐ON RF, which was typical of the majority of the responding monocular cells (*n* = 13/19 cells; of the remaining neurons 5 had contralateral‐OFF RFs and 1 had detectable ON responses only when both eyes were viewing).

By contrast to the above, a much larger subset of the cells classified as ‘binocular’ exhibited detectable RFs with the visual display position as in Fig. 8 *A* (*n* = 11/14; ∼79%). This proportion is similar to that for binocular cells in the dLGN tested with the same stimulus (Howarth *et al*. 2014) and approximately corresponds to the fraction of the binocular visual zone covered by our visual display. Also in common with our previous analysis of dLGN neurons, typically these cells exhibited a strong preference towards one of the two eyes (*n* = 8/11 cells where we could map an RF). In the example shown in Fig. 8 *C* (which responded to the appearance of light but not dark bars), there was a strong ipsilateral‐ON RF and a weaker contralateral‐ON RF, but across the sample we found equal numbers of contralateral and ipsilateral biased neurons (*n* = 4 for both; the remaining 3 cells only exhibited quantifiable RFs when we presented bars with both eyes able to view the stimulus).

Most importantly here, we were surprised to find (given the data for binocular neurons reported above) that cells with antagonistic responses very rarely exhibited detectable changes in firing rate following the appearance of bars in the binocular visual zone (Fig. 8 *A*). Indeed, 0/7 ipsi‐ON cells and only 2/14 contra‐ON antagonistic cells responded to the appearance of bar stimuli under any of the three viewing conditions tested here (i.e. contralateral‐only, ipsilateral‐only or both eyes). These proportions of responding antagonistic cells were significantly lower than that for binocular cells where we could map RFs (Fig. 8 *A*, Fisher's exact test: *P* = 0.001 and *P* = 0.002 *vs*. ipsi‐ON and contra‐ON antagonistic cells). By contrast, the proportion of antagonistic cells with detectable RFs was in fact equivalent to that of monocular cells with RFs within regions of space visible to both eyes (Fig. 8 *A*; Fisher's exact test, *P* = 0.59 and *P* > 0.99 for ipsi‐ON and contra‐ON antagonistic cells, respectively).

Of the two contra‐ON/ipsi‐OFF cells that did respond to bar stimuli, both lacked any detectable responses to stimuli presented to the ipsilateral eye but exhibited clear ON excitatory RFs when stimuli were presented exclusively to the contralateral eye (Fig. 8 *D*; cell exhibits increases in firing for white bars and decreases in firing for black bars). Moreover, when bars were presented with both eyes viewing, ON RFs persisted in the same region of visual space (Fig. 8 *D* and *E*). Overall, then, this behaviour was similar to that of the monocular cells described previously (Fig. 8 *B*). We also note here that while both of the two contra‐ON/ipsi‐OFF cells we detected exhibited modestly reduced response amplitude when bars were presented under binocular *vs*. contralateral‐only viewing conditions (33% and 42% reductions for the two cells), this was within the range observed for monocular cells (−72% reduction to 52% increase for the 18 cells that responded under both conditions). We suspect this variation reflects changes in cell responsiveness over time (e.g. due to contrast adaptation), since each viewing condition was tested sequentially. Importantly, however, other RF parameters (position and width) were very consistent under monocular and binocular viewing conditions for all cell groups identified here (Fig. 8 *E*; *r* = 0.94, 0.92 and 0.88 for RF azimuth, elevation and radius, respectively). These data indicate, therefore, that changes in eye position did not impact our analyses.

Since we were unable to define ipsilateral RFs for any of the antagonistic cells within the binocular zone, in a subset of the experiments described above (*n* = 7) we also moved the monitor laterally to cover large regions of the ipsilateral visual field (outside the binocular zone). As expected, none of 48 monocular or six binocular IGL/vLGN neurons encountered in these experiments responded to bar stimuli presented to the lateral ipsilateral field. More importantly, nor did any of the six antagonistic IGL/vLGN neurons we identified (4 contra‐ON and 2 ipsi‐ON). Based on the portions of the full ipsilateral visual field we tested (∼33.3% for these 7 experiments and 16.7% for the remaining 17 experiments in which we did not reposition the screen), if antagonistic cells did have discrete RFs originating from the ipsilateral eye, the chance of not identifying one from 21 cells is very low (*P* = 0.006; assuming a random distribution across space). On that basis, we suspect that ipsilateral components of the antagonistic cell responses are insensitive to the stimuli we applied here, either because these require relatively global changes in irradiance, or because their responses are too slow to detect.

**Figure 9:**
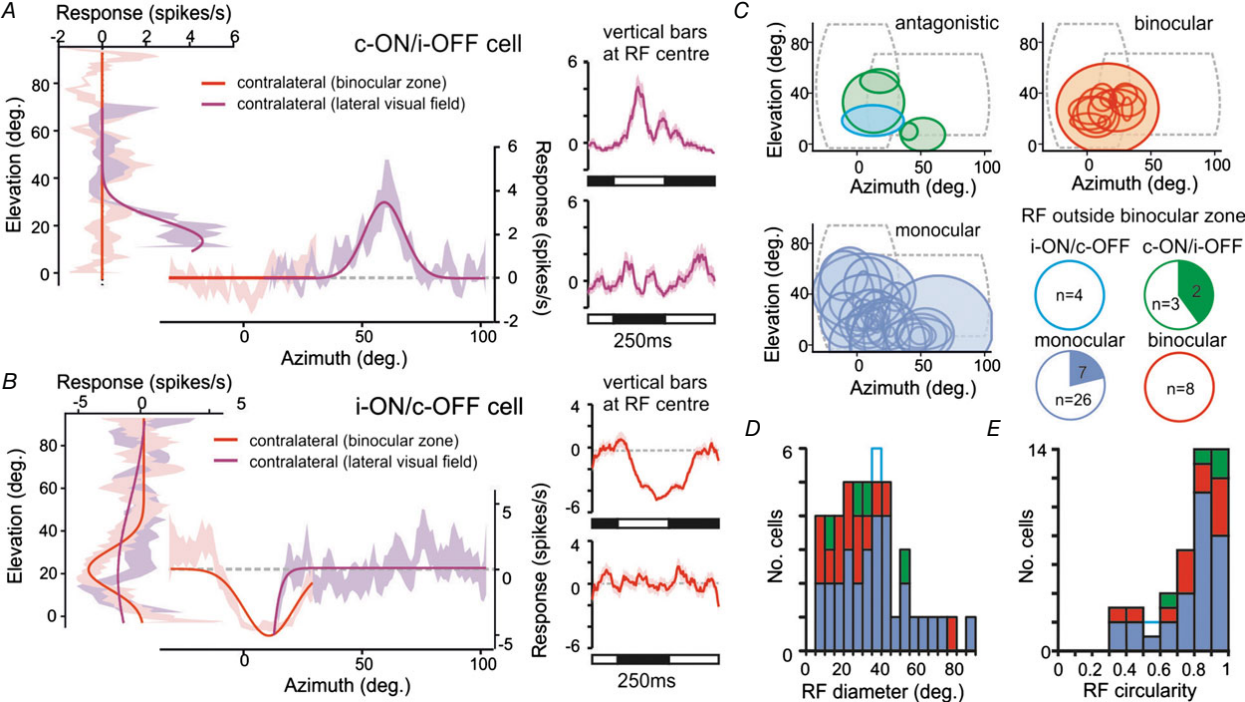
Contralateral retinal receptive fields for antagonistic cells resemble those of monocular IGL/vLGN neurons *A* and *B*, responses of contra‐ON/ipsi‐OFF (*A*) and ispsi‐ON/contra‐OFF (*B*) antagonistic cells whose contralateral driven RFs were mapped with a monitor placed in the binocular visual zone and lateral contralateral visual field (conventions otherwise as in Fig. 8 *B–D*). Note that for cell in *A* the receptive field lies outside the region of space visible to both eyes. *C*, RF positions and sizes for all responding neurons identified in this study (*n* = 4 contra‐ON/ipsi‐OFF; *n* = 1 ipsi‐ON/contra‐OFF; *n* = 13 binocular cells, *n* = 30 monocular cells). Dashed lines in each panel show the regions of visual space occupied by the display at the two positions tested. Pie charts show proportion of tested neurons from each class exhibiting contralateral RFs outside of regions of space visible to both eyes. *D* and *E*, RF diameter (*D*) and circularity (ratio of long:short axis) (*E*) for all RFs mapped in this study (sample sizes as above).

In sum, our data suggest that antagonistic cells do not preferentially respond to visual contrast (of the type we provided) within regions of space visible to both eyes. To further test this possibility, we performed additional experiments in which we specifically evaluated contralateral RFs both within the binocular visual zone and also within portions of the visual field only visible to the contralateral eye. In these experiments (*n* = 8), we identified five cells with contra‐ON/ipsi‐OFF responses, two of which exhibited ON RFs outside of the region of space that is visible to the ipsilateral eye (the remaining three cells were unresponsive across all regions tested). The responses of one of those two responding antagonistic cells is shown in Fig. 9 *A*, illustrating a contralateral ON RF field centred at an angle of ∼60° from the midline (field of view for the ipsilateral eye is at most 30° into contralateral visual space).

Importantly, the proportion of contra‐ON/ipsi‐OFF cells in which we could identify RFs outside of the binocular zone (*n* = 2/5 cells tested) was equivalent to that for monocular neurons recorded in the same experiments (Fig. 9 *C*; *n* = 7/33 cells tested; Fisher's exact test, *P* = 0.570). By contrast, none of the eight binocular cells recorded in these experiments had RF centres outside the binocular zone (Fig. 9 *C*).

We also identified four ipsi‐ON/contra‐OFF antagonistic cells in these recordings, one of which exhibited a clear contralateral OFF RF that was located within the binocular visual zone (Fig. 9 *B*). Unfortunately, this particular set of experiments did not include ipsilateral or binocular viewing conditions. Nonetheless, this finding established that at least some ipsi‐ON/contra‐OFF cells exhibited a defined contralaterally driven RF, while the low proportion of such cells identified across all experiments (*n* = 1/11) indicates that either the RFs of such cells are not preferentially located within the binocular zone or are primarily responsive to different stimuli than those employed here.

Figure 9 *C* also illustrates the size and position of all contralateral RFs recorded in this study, including those described above and those for cells contributing to Fig. 8. Taking all those data into account, while it is hard to draw definitive conclusions regarding ipsi‐ON/contra‐OFF cells, it seems the RFs of contra‐ON/ipsi‐OFF cells exhibit very similar properties to those typical of monocular neurons in the IGL/vLGN. Contralateral RFs tend to be large (Fig. 9 *D*; 14–50° diameter for the 4 contra‐ON/ipsi‐OFF cells; median ± SD for monocular cells = 37 ± 19°), circular (Fig. 9 *E*; contra‐ON/ipsi‐OFF aspect ratio = 0.67–0.93; median ± SD for monocular cells = 0.83 ± 0.19) and are either randomly distributed across the contralateral visual field or are certainly not preferentially located within the binocular zone (Fig. 9 *C*).

## Discussion

Here we show that commissural signalling plays a fundamental role in determining the sensory properties of a specific subset of IGL/vLGN neurons that exhibit opposing responses to light driven via either eye. Specifically, we demonstrate that commissurally projecting IGL/vLGN neurons primarily convey signals originating with crossed retinal inputs and IGL/vLGN neurons receiving excitatory or inhibitory commissural input respectively exhibit ON or OFF responses driven by the ipsilateral eye. Moreover, we go on to show that the antagonistic responses of these neurons rely on commissural input, since they are abolished by unilateral inactivation of the contralateral visual thalamus. Below, we consider in detail the organisation and properties of the circuits responsible for generating these antagonistic responses and then their significance for sensory coding.

In the case of IGL/vLGN cells with contra‐ON/ipsi‐OFF type antagonistic visual responses, our data support a relatively straightforward origin (Fig. 5). These cells exhibit inhibitory responses to stimulation of the contralateral LGN and the ipsilateral‐OFF component of their visual responses is abolished by inactivation of the opposing LGN, leaving a pure contralateral ON response. As such, we infer that this latter component arises via a direct retinal projection while the ipsilateral component arises via inhibitory inputs from cells in the opposite IGL/vLGN that receive crossed retinal projections. Several lines of evidence support this view: (1) much of the retinal input to the IGL and dorsal/central portions of the vLGN arises via melanopsin‐expressing retinal ganglion cells (RGCs) which show exclusively sustained ON responses (Chen *et al*. 2011; Zhao *et al*. 2014); (2) the majority of mouse IGL/vLGN neurons exhibit pure contralateral ON responses, including those antidromically activated from the opposite hemisphere (present study; Howarth *et al*. 2014; Hanna *et al*. 2017); (3) IGL/vLGN neurons are primarily GABAergic (Harrington, 1997) and many previous studies in rodents, including mice (Cosenza & Moore, 1984; Mikkelsen, 1992; Park *et al*. 1993; Morin & Blanchard, 1998; Moore *et al*. 2000; Vrang *et al*. 2003; Oh *et al*. 2014), reveal robust input to the IGL/vLGN region from its counterpart in the opposite hemisphere.

In line with the idea that contralateral components of the contra‐ON/ipsi‐OFF cell responses arise via direct retinal projections, we were able to map contralateral ON RFs for a subset of these cells, the properties of which were similar to those of conventional IGL/vLGN monocular cells and those reported previously for rat IGL/vLGN neurons (Hale & Sefton, 1978). Of particular note, the contralateral RFs of IGL/vLGN neurons were ∼2–3 times larger (often > 30°) than in other visual thalamic nuclei (Grubb & Thompson, 2003; Piscopo *et al*. 2013; Howarth *et al*. 2014; Allen *et al*. 2016), suggesting convergent input from multiple RGCs.

Most significantly here, however, our data indicated that the contralateral RFs of the contra‐ON/ipsi‐OFF cells were rarely identifiable in portions of visual space visible to both eyes. In principle, it is possible that this simply reflects the fact that our stimuli were suboptimal for evoking responses from these neurons. However, the fact that we were able to identify RFs for some of these cells (including two that lay outside binocular visual space) leads us to suspect that instead the RFs for these cells are distributed across the contralateral visual field (including regions that we did test). As such, we infer that integration of eye‐specific signals in these contra‐ON/ipsi‐OFF cells does not operate in the same way it does for LGN cells with conventional binocular visual responses (Grieve, 2005; Howarth *et al*. 2014; Zeater *et al*. 2015).

It is also noteworthy that we were unable to map a discrete ipsilateral RF for any of the contra‐ON/ipsi‐OFF cells described here. At present we cannot definitively resolve whether this is because our stimulus properties were not well matched to the ipsilateral RFs of such cells or whether this is because the ipsilateral response requires more global changes in irradiance (e.g. conveyed by convergent input from multiple commissurally projecting neurons). Nonetheless, we think it unlikely that these cells have ipsilateral RFs that are tuned to movement or very small stimuli since full field light steps evoked readily measurable changes in firing from these cells. We did, however, note that contrast‐dependent components of contra‐ON/ipsi‐OFF cell responses were strongly biased towards the contralateral retina (Fig. 6). It is therefore formally possible that these cells do in fact have a discrete ipsilateral RF (albeit covering a portion of visual space that doesn't necessarily overlap with the contralateral RF) which would become visible under prolonged visual stimulation.

Importantly, even if this latter possibility were true, given that the image projected onto the retina is never stationary for long (due to eye/body or externally generated movement) such sluggish kinetics would be expected to substantially reduce any spatial information provided. As such, it appears that contra‐ON/ipsi‐OFF cells multiplex two kinds of visual information, conventional visual contrast responses driven by the contralateral retina and information about interocular differences in irradiance, encoded in their baseline firing rate.

In the case of antagonistic cells with ipsi‐ON/contra‐OFF responses, our data suggest a related (albeit somewhat more complex) circuitry to that described above for the other kind of antagonistic cell (Fig. 5). A number of facets of our data are worthy of specific discussion, however. Firstly, ipsi‐ON/contra‐OFF cells show excitatory responses to stimulation of the contralateral IGL/vLGN, with a latency consistent with a monosynaptic projection. Previous work in rats identified a subset of IGL neurons whose firing rate increased following intense electrical stimulation of the contralateral thalamus (Zhang & Rusak, 1989). The identification of fast excitatory responses here is nonetheless a little surprising since the IGL/vLGN is predominantly GABAergic, and both GABA and key neuropeptides expressed by IGL neurons (NPY and ENK) appear to exert purely inhibitory effects in this region (Palus *et al*. 2015, 2017). A small population of cells in the IGL/vLGN express vesicular glutamate transporters (experiment: 73818754; Lein *et al*. 2007), however, suggesting the presence of some glutamatergic neurons that could account for the excitatory responses we observed.

A second key point relates to the precise nature of the sensory signals ipsi‐ON/contra‐OFF cells receive from the contralateral hemisphere. In common with their counterparts with the opposite response preference, ipsilateral responses of this group of cells were supressed following thalamic inactivation. Coupled with the data from our electrical stimulation experiments, this indicates that ipsi‐ON/contra‐OFF cells receive excitatory signals from cells in the opposite hemisphere whose responses include a contralateral ON component. We also, however, found that the thalamic inactivation reversed the contralateral‐OFF component of this group of antagonistic cells (converting it to an ON excitation). Presumably such cells also then receive some weak contralateral ON input from the retina while the commissural inputs they receive also includes cells that are responsive to ipsilateral retinal signals, i.e. excitatory input from cells with ipsi‐OFF responses or inhibitory input from cells with ipsi‐ON responses.

The only IGL/vLGN neurons identified here or (to our knowledge) previously (Harrington & Rusak, 1989; Howarth *et al*. 2014) with ipsilateral OFF responses are the contra‐ON/ipsi‐OFF antagonistic cells. We did not identify any contra‐ON/ipsi‐OFF neurons via antidromic activation, but since such cells are quite rare (<10% of all visually responsive neurons we identified in the IGL/vLGN) we cannot definitively rule out the possibility that some do provide commissural projections. By contrast, while cells with pure ipsi‐ON responses seem to be absent from the IGL/vLGN (Harrington & Rusak, 1989; Howarth *et al*. 2014), we do find cells with binocular excitatory responses, some of which can be antidromically activated from the contralateral hemisphere.

Together, then, two possible explanations for our thalamic inactivation data are that (1) ipsi‐ON/contra‐OFF cells receive excitatory input from contra‐ON/ipsi‐OFF cells in the opposite hemisphere or (2) ipsi‐ON/contra‐OFF responses involve commissural inputs from excitatory cells with monocular (contralateral ON) responses and inhibitory cells with binocular ON responses that include a strong ipsilateral component. We favour this latter explanation (Fig. 5) since the expected response properties better fit those observed in our other experiments. Firstly, ipsi‐ON/contra‐OFF cells had more closely matched eye‐specific contrast responses (Fig. 7) compared to those predicted from contra‐ON/ipsi‐OFF cell input. Secondly we were able to map a contralateral‐OFF RF for one of these antagonistic cells and strong ipsilateral RFs were observed in some binocular neurons but never in contra‐ON/ipsi‐OFF cells (Fig. 8). In any case, while our present experiments do not definitively distinguish between the possible mechanisms outlined above, it is clear from our data that commissural input is essential for the cell to express antagonistic type responses.

At present we can only speculate as to the functional significance of IGL/vLGN neurons with antagonistic binocular responses. What is clear is that neither type of antagonistic cell possesses discrete ipsi‐ and contralateral RFs that occupy overlapping regions of visual space and exhibit equivalent sensory properties (as for more conventional binocular LGN cells). Rather, while we do find evidence for discrete contralateral RFs among both classes of cells, it seems that ipsilateral response components may operate over different spatial and/or temporal scales. As such, it seems such neurons would be most active when a region of space covered by their contralateral RF is substantially brighter or dimmer than the average amount of light reaching the ipsilateral eye. Such a mechanism could serve to highlight especially salient features of the visual scene for the purpose of directing eye movements – a putative role of the IGL/vLGN (Harrington, 1997; Morin & Allen, 2006). Similarly, such signals might potentially provide useful information for vestibular processing (Horowitz *et al*. 2004), insofar as one would predict antagonistic cell activity to be strongly modulated when head/body movements direct one eye towards the sky and the other towards the ground.

In addition to the putative functions described above, the best known role for the IGL is in regulation of the circadian system (Morin & Allen, 2006; Brown, 2016). A previous study in hamster identified one cell with contra‐ON/ipsi‐OFF responses that projected to the SCN, suggesting such cells may play some specific role in regulation of circadian photoentrainment (Harrington & Rusak, 1989). Our previous work did not reveal any substantive population‐level changes in ipsilateral SCN visual responses following inhibition of geniculohypothalamic signalling (Hanna *et al*. 2017). Nonetheless, since at the single neuron level we find considerable diversity in eye specific response properties across the SCN (Walmsley & Brown, 2015), we do not rule out the possibility of a contribution from antagonistic cells to the responses of some SCN neurons. Since geniculohypothalamic signals act to reduce SCN responses to retinal input (Hanna *et al*. 2017), antagonistic cells could be useful in this context so as to attenuate aberrant circadian responses to anomalously bright elements of the visual scene (e.g. direct viewing of the setting/rising sun).

Related to the above, since the IGL/vLGN also receive some input from the SCN (Morin & Allen, 2006), we should consider the possibility that antagonistic responses are themselves under circadian control. As far as we are aware, the possibility of circadian modulation of IGL/vLGN visual responses has yet to be investigated in detail. However, we recently investigated spontaneous and evoked IGL/vLGN activity in an *ex vivo* prep that retains geniculohypothalamic connectivity and did not find any clear evidence for coordinated circadian variation in responses to optic tract input (Hanna *et al*. 2017). On balance, then, we think it unlikely that the proportion of cells showing antagonistic responses or their sensory properties would exhibit significant circadian variation. However, since in the present study all our recordings were performed during the projected day phase, future work would be required to definitively resolve this question.

A final point to note here is that, while in the present study we focused on antagonistic cells in the IGL/vLGN, our data using electrical stimulation also reveal the presence of some cells with excitatory or inhibitory binocular responses that receive commissural signals. Our thalamic inactivation experiments did not imply a major role for commissural signalling in dictating sensory preferences among cells with excitatory binocular responses (while cells with inhibitory responses were sufficiently rare that we did not identify any in this particular set of experiments). Nonetheless, on balance these data suggest that the role of commissural communication in the visual thalamus is unlikely to be exclusively limited to generating antagonistic eye‐specific responses.

In conclusion, our data provide new insight into the significance of IGL/vLGN commissural communication, revealing a key role in generating the characteristic sensory properties of neurons with antagonistic binocular responses. In turn, the information about interocular difference in irradiance provided by such cells potentially offers a useful new dimension of sensory control to functions involving the IGL/vLGN including regulation of the circadian, visuomotor and vestibular systems.

## Additional information

### Competing interests

The authors declare no competing financial interests.

### Author contributions

A.P., L.W., E.H. and M.H. performed experiments. A.P., L.W., E.H. and T.M.B. analysed the data. T.M.B. designed the experiments and wrote the manuscript with assistance from all other authors. All authors have read and approved the final version of this manuscript and agree to be accountable for all aspects of the work in ensuring that questions related to the accuracy or integrity of any part of the work are appropriately investigated and resolved. All persons designated as authors qualify for authorship, and all those who qualify for authorship are listed.

### Funding

This work was supported by a Biotechnology and Biological Sciences Research Council (UK) awards to T.M.B (BB/I017836/1 and BB/N007115/1) and strategic skills award to L.W.
